# Targeting TET3 in macrophages provides a concept strategy for the treatment of endometriosis

**DOI:** 10.1172/JCI185421

**Published:** 2024-11-01

**Authors:** Hossein Hosseinirad, Md Saidur Rahman, Jae-Wook Jeong

**Affiliations:** Department of Obstetrics, Gynecology and Women’s Health, University of Missouri, Columbia, Missouri, USA.

## Abstract

Endometriosis, characterized by the presence of endometrial-like tissue outside the uterus, is a condition associated with pain and infertility. In this issue of the *JCI*, Lv et al. illuminate the critical pathophysiological role of the ten-eleven translocation 3 (TET3) in endometriosis. TET3 expression levels were higher in macrophages of endometriotic lesions compared with control endometrial tissue, implicating TET3 as a contributing factor in the chronic inflammation that occurs in endometriosis. TGF-β1 and MCP1 are present in the peritoneal cavity of women with endometriosis, and macrophage exposure to these factors resulted in upregulation of TET3, thereby promoting their survival. Notably, Bobcat339, a selective TET inhibitor, induced apoptosis in these macrophages. Further, myeloid-specific TET3 loss reduced endometriosis in mice. RNA-Seq analysis following TET3 knockdown revealed alterations in cytokine signaling and cell-death pathways, underscoring the therapeutic potential of targeting TET3 in macrophages as a strategy for managing endometriosis.

## Macrophages overexpress TET3 in endometriosis

Macrophages are key immune cells in inflammation and tissue repair that contribute to the pathogenesis of endometriosis. Proinflammatory macrophages are actively recruited to endometriotic lesions, where they exacerbate to the local inflammatory environment by secreting cytokines, growth factors, and other proinflammatory mediators (1). Their altered function in endometriosis promotes angiogenesis, tissue remodeling, and resistance to immune clearance, thereby perpetuating the condition’s chronic nature (1).

Ten-eleven translocation 3 (TET3), a member of the TET enzyme family, plays a pivotal role in regulating DNA methylation and gene expression by converting 5-methylcytosine to 5-hydroxymethylcytosine (2). Chronic overexpression of TET3 is linked to various inflammatory conditions, including type 2 diabetes and liver fibrosis, suggesting a broader involvement of TET3 in inflammatory processes (2). In this issue of the *JCI*, Lv et al. (3) demonstrate an association between TET3-overexpressing macrophages and inflammation in endometriosis. The authors used rigorous methodologies including single-cell RNA-Seq (scRNA-Seq) data analysis, genetic and pharmacological approaches, and both in vitro and in vivo models facilitating a thorough examination of TET3’s role in macrophages. Specifically, they observed an eight-fold increase in TET3-overexpressing macrophages in peritoneal endometriotic lesions compared with healthy endometrial tissue, emphasizing TET3’s role in the disease’s inflammatory milieu.

Macrophages are highly adaptable cells that modify their functions in response to environmental signals (4). Lv et al. demonstrated that factors such as TGF-β1 and MCP1, which are elevated in the peritoneal cavities of patients with endometriosis, can upregulate TET3 in macrophages. Additionally, conditioned media from endometriotic stromal cells further enhanced TET3 expression, an effect that was mitigated by TGF-β1–specific antibodies. These findings indicate that TET3 is dynamically regulated by the endometriotic microenvironment, affecting macrophage function and survival. Moreover, TET3 knockdown induced apoptosis, further indicating TET3 as a crucial factor for macrophage viability (3).

## TET3-overexpressing macrophages promote inflammation in endometriosis

**Figure 1:**
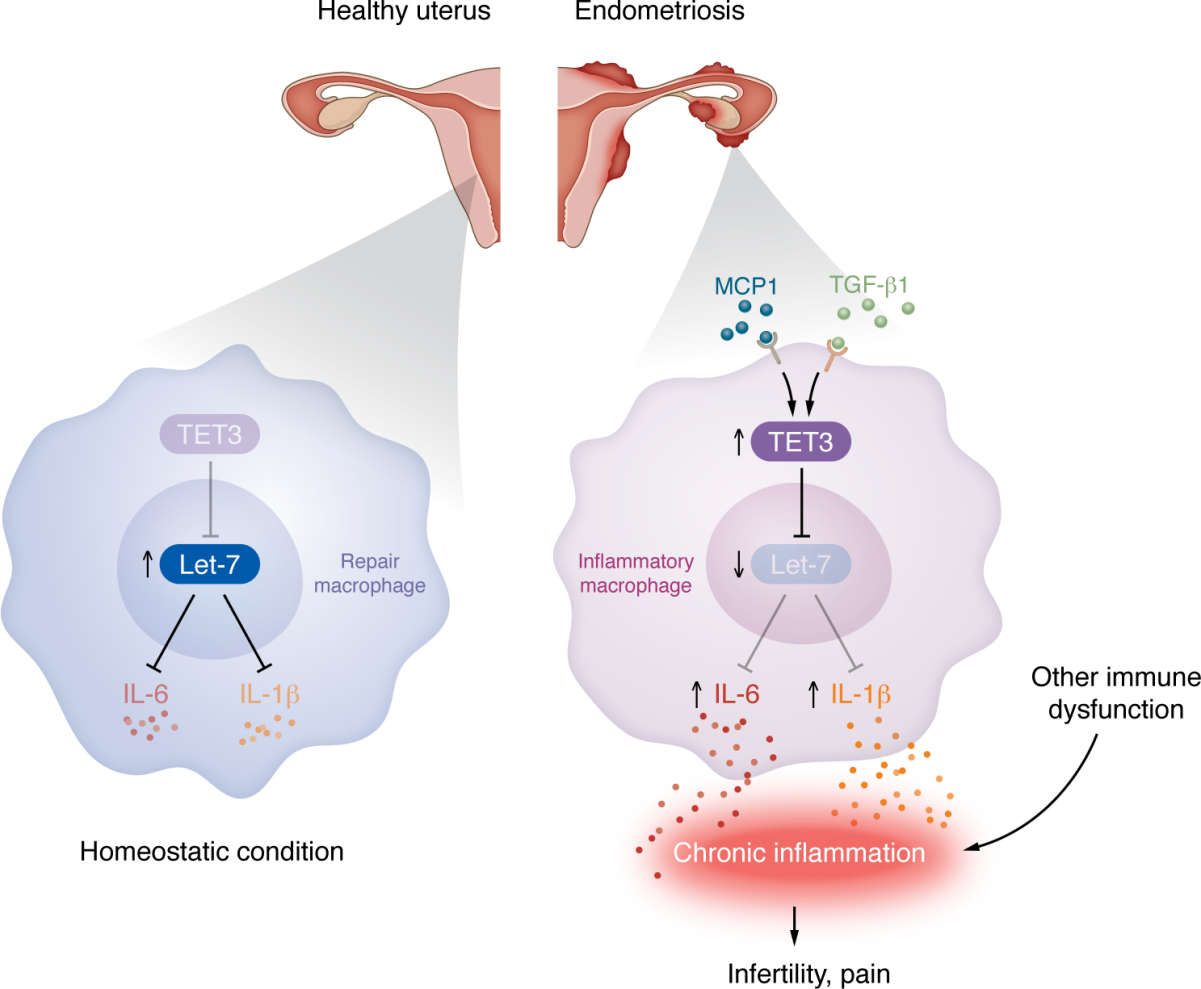
Macrophage-expressed TET3 has a pathophysiological role in endometriosis. In a healthy uterine environment, antiinflammatory, repair macrophages produce interleukins and interferons that maintain healthy tissue. In contrast, inflammatory macrophages associated with endometriotic lesions express increased TET3 levels. The endometriosis environment has increased levels of TGF-β1 and MCP1, both of which can induce TET3 expression in macrophages. TET3 blocks the posttranscriptional miRNA regulator let-7, resulting in increased production and secretion of the proinflammatory cytokines IL-6 and IL-1β. TET3 also interacts with the STAT3/NCOR1/HDAC4 transcriptional corepressor complex to exert additional inflammatory effects.

RNA-Seq analysis revealed that TET3 knockdown particularly changed pathways related to cytokine/chemokine signaling and cell death. There was an upregulation of proapoptotic genes such as *Bcl2l11*, *Bid*, and *Pmaip1* alongside alterations in genes involved in inflammatory responses. Notably, the modulation of IL-6 expression in response to TET3 levels underscores its impact on macrophage function and inflammation (3). IL-6 is crucial for macrophage polarization and survival, influencing the broader inflammatory landscape of endometriosis (5, 6). Elevated IL-6 levels are often associated with increased macrophage activity and chronic inflammation, further linking TET3 with disease pathology. The interplay between TET3 and IL-6 suggests that TET3-overexpressing macrophages may drive the inflammatory response in endometriosis by modulating IL-6 signaling (Figure 1). Understanding this relationship provides insight into how TET3 contributes to the disease’s inflammatory environment and highlights potential therapeutic targets.

TET enzymes, particularly TET3, play a crucial role in modulating the gene-expression profiles of macrophages through their epigenetic modifications. This capability enables macrophages to rapidly adapt to changes in their environment, a vital function given their role in immune responses (7). Unlike other cell types, macrophages exhibit remarkable plasticity, allowing them to continuously adjust their functional states between proinflammatory and antiinflammatory phenotypes in response to local stimuli. This dynamic modulation is essential for effective immune responses and tissue repair, as macrophages must respond appropriately to varying signals encountered during inflammation and injury (7). Within this context, TET3 emerges as a pivotal regulator of macrophage gene expression, underscoring its importance in the broader framework of immune adaptability.

A central component of this regulatory network is IL-6, a proinflammatory cytokine that becomes activated in chronic disease states (8). Lv et al. have shown that TET3 overexpression in macrophages leads to increased IL-6 levels, which are mediated by the inhibition of let-7 microRNAs (3). Upon stimulation, IL-6 activates the JAK/STAT3 signaling pathway, facilitating the recruitment of STAT3 to specific gene promoters, including those that regulate IL-6 and other inflammatory mediators (8, 9). This interaction enhances the transcription of genes associated with inflammation, thereby facilitating the transition of macrophages to a proinflammatory state. Moreover, TET3 has been shown to interact directly with STAT3, further complicating the regulatory mechanisms at play (10). This direct interaction suggests that TET3 may influence gene expression indirectly through IL-6 and enhance the transcriptional activity of STAT3 itself, amplifying the inflammatory response.

Thus, the interplay among TET3, IL-6, and STAT3 is vital for maintaining the balance between proinflammatory and antiinflammatory macrophage states. This balance is crucial for effective immune regulation, as dysregulation of these pathways can lead to persistent inflammation and contribute to various inflammatory diseases including endometriosis. Understanding the intricate relationships among TET3, IL-6, and STAT3 not only provides valuable insights into the molecular mechanisms underlying macrophage function, but also highlights potential therapeutic targets for modulating immune responses, offering hope for the future of immune-response modulation.

## Clinical implications and considerations

Lv et al.’s use of human and mouse models enhances the robustness and applicability of the findings, providing strong translational potential. Targeting TET3 offers a promising strategy to modulate the inflammatory environment in endometriosis. Bobcat339 is a synthetic molecule that destabilizes TET3 (3). Particularly important was the finding that Bobcat339 selectively degraded TET3 and reduced endometriosis progression in mice. Bobcat 339 also promoted apoptosis in TET3-overexpressing macrophages via von Hippel-Lindau (VHL) E3 ubiquitin ligase-mediated degradation. These findings emphasize the importance of TET3 in macrophage function and offer a treatment strategy for endometriosis and possibly other related inflammatory conditions. However, the complexity of TET3’s involvement across different cell types calls for more research to fully understand its potential side effects and to develop targeted therapies.

Despite numerous strengths of Lv et al., some areas for improvement exist. The reliance on a single marker (CD163) to identify macrophages in human and mouse tissues, for example, might not fully capture the heterogeneity within macrophage populations and could overlook other influential subpopulations (11). Additionally, Lv et al. (3) did not address the potential variability in TET3 expression and macrophage behavior across different stages of endometriosis or among different patient demographics, which could impact the generalizability of the findings. Another methodological concern involved the use of the LysM-cre strain for myeloid-specific *Tet3* knockout, as LysM is also expressed in other myeloid lineage cells, including granulocytes and dendritic cells (12, 13). This crossreactivity could introduce confounding factors that obscure the specific effects of macrophage-specific TET3 loss. Furthermore, while the study demonstrated the therapeutic potential of Bobcat339, it did not thoroughly explore the potential off-target effects and long-term safety of this compound, which are crucial for clinical translation. Preclinical studies focusing on the pharmacokinetics, pharmacodynamics, and toxicity profiles of such compounds will be essential for advancing them toward clinical trials.

Overall, Lv et al. (3) provides valuable insights into the role of TET3-overexpressing macrophages in endometriosis, highlighting their effects on inflammation and the microenvironment. The interplay between TET3 and IL-6 particularly underscores the importance of targeting TET3 in macrophages as a promising strategy to improve outcomes for patients with endometriosis. We look forward to further investigations that explore the clinical implications of TET3-targeted therapies.

## Version 1 11/01/2024

Electronic publication
